# Isolation and Identification of Free-Living Amoebae from Tap Water in Sivas, Turkey

**DOI:** 10.1155/2013/675145

**Published:** 2013-07-22

**Authors:** Kübra Açıkalın Coşkun, Semra Özçelik, Lütfi Tutar, Nazif Elaldı, Yusuf Tutar

**Affiliations:** ^1^Department of Parasitology, Faculty of Medicine, Cumhuriyet University, 58140 Sivas, Turkey; ^2^Department of Biology, Faculty of Science and Letters, Kahramanmaraş Sütçü İmam University, 46100 Kahramanmaras, Turkey; ^3^Department of Infectious Diseases, Faculty of Medicine, Cumhuriyet University, 58140 Sivas, Turkey; ^4^Department of Biochemistry, Faculty of Pharmacology, Cumhuriyet University, 58140 Sivas, Turkey; ^5^CUTFAM Research Center, Faculty of Medicine, Cumhuriyet University, 58140 Sivas, Turkey

## Abstract

The present work focuses on a local survey of free-living amoebae (FLA) that cause opportunistic and nonopportunistic infections in humans. Determining the prevalence of FLA in water sources can shine a light on the need to prevent FLA related illnesses. A total of 150 samples of tap water were collected from six districts of Sivas province. The samples were filtered and seeded on nonnutrient agar containing *Escherichia coli* spread. Thirty-three (22%) out of 150 samples were found to be positive for FLA. The FLA were identified by morphology and by PCR using 18S rDNA gene. The morphological analysis and partial sequencing of the 18S rDNA gene revealed the presence of three different species, *Acanthamoeba castellanii*, *Acanthamoeba polyphaga*, and *Hartmannella vermiformis*. *Naegleria fowleri*, *Balamuthia mandrillaris*, or *Sappinia* sp. was not isolated during the study. All *A. castellanii* and *A. polyphaga* sequence types were found to be genotype T4 that contains most of the pathogenic *Acanthamoeba* strains. The results indicated the occurrence and distribution of FLA species in tap water in these localities of Sivas, Turkey. Furthermore, the presence of temperature tolerant *Acanthamoeba* genotype T4 in tap water in the region must be taken into account for health risks.

## 1. Introduction

Free-living amoebae (FLA), ubiquitous and widely distributed protozoa, feed on bacteria, algae, fungi, and small organic particles and are adaptable to their environment [1]. They can be found in dust, air, seawater, dental treatment units, sewage, eyewash solutions, contact lenses, and dialysis units and are particularly abundant in soil and water [2, 3]. Among them, only four genera including *Acanthamoeba*, *Naegleria*, *Balamuthia*, and *Sappinia* cause opportunistic and nonopportunistic infections in humans and in animals, but infections are not commonly reported with the exception of *Acanthamoeba keratitis *which is reported in over 1 to 2 cases per million contact lens wearers in the USA annually [4–6]. *Acanthamoeba* spp. and *Balamuthia mandrillaris *cause granulomatous amoebic encephalitis (GAE) while *Naegleria fowleri *causes primary amoebic meningoencephalitis (PAM). Both GAE and PAM are central nervous system infections. Some *Acanthamoeba *spp., commonly *Acanthamoeba castellanii*, cause amoebickeratitis (AK), a vision-threatening corneal infection. In humans *Acanthamoeba* spp. may also affect the skin and lungs [3, 7]. *Hartmannella* spp. invade animal tissues and have been found in nasal mucosa of humans, the bronchial system of dogs, and the intestines of turkeys [8]. *Sappinia diploidea* have been reported, only once, from a brain infection in a healthy man [9]. This amoeba was identified later as *Sappinia pedata*, by using real-time PCR tests based on 18S rRNA gene sequences [10].

The presence of FLA in tap water may represent a health risk to both immunocompromised and immunocompetent individuals [11] and they are resistant to extreme conditions of temperature, pH, and exposure to various chemicals [2, 5]. In addition to their pathogenicity, FLA serve as hosts for a large number of pathogenic bacteria and viruses for humans including *Legionella* spp., *Vibrio cholerae*, *Burkholderia cepacia, Listeria monocytogenes, Escherichia coli O157, Mycobacterium *spp., *Coxsackievirus, Adenovirus*, and* Echovirus *[2, 5, 11]. Furthermore, FLA can increase virulence of some of the bacteria called amoeba-resisting microorganisms (ARMs) including *Mycobacterium* spp., *Pseudomonas aeruginosa*, *Legionella* spp., *Cryptococcus neoformans*, and *Histoplasma capsulatum* [11].

An increase in the number of intracerebral infections caused by FLA in the USA and worldwide has been reported [6]. 

FLA human infections are documented [12–14], but limited information is available in the literature concerning FLA from the environmental samples in Turkey [15–17]. Therefore, the aim of this study is to isolate FLA from tap water samples collected from various districts in the province of Sivas by employing morphological and molecular methods in order to contribute to the study of understanding their ecology and to identify any potential health risks.

## 2. Subjects and Methods

### 2.1. Study Location, Sampling, Isolation, and Identification of Amoebae and DNA Extraction

**Figure 1:**
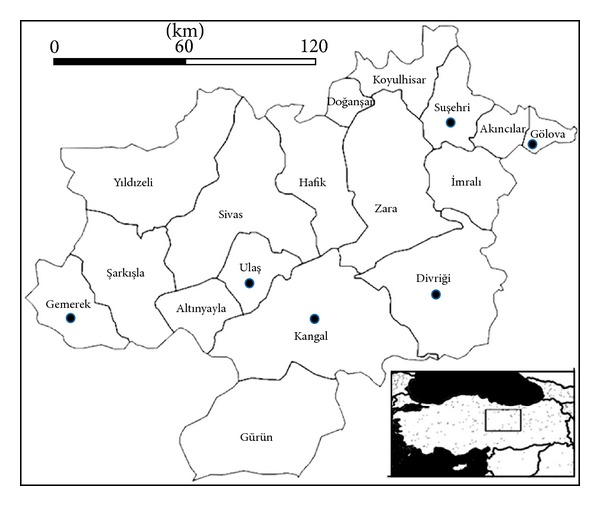
Districts of Sivas province and water sample locations (dots), Turkey.

A total of 150 tap water samples were collected between March and August 2011 from the districts of Divriği, Kangal, Suşehri, Ulaş, Gölova, and Gemerek of Sivas province, located in the central Anatolia region of Turkey. The total surface area of Sivas province is 28500 km^2^ and the study area is about 10000 km^2^ (Figure 1). Sivas is located at the junction of different regions and reflects typical climates of Turkey's various regions. Therefore, a prevalence study in a transition region like Sivas may reflect overall Turkish FLA distribution.

All samples (except two of them, see Table 2) in this study were chlorinated by the city officials in drinking water plant according to world health organization criteria (0.2–0.7 ppm).

A total of 500 mL of water sample was collected from each tap focus in a sterile plastic container from different villages and districts. They were then immediately transferred to the laboratory. FLA were isolated from the samples as previously described elsewhere [7, 18]. Briefly, water samples were filtered through 0.45 *μ*m pore size cellulose nitrate membrane filter (47 mm in diameter) under a vacuum. The membrane filters for each water sample were scraped and collected materials were placed in 15 mL of sterile cover tubes containing 10 mL phosphate buffered saline (PBS). Tubes were incubated at room temperature overnight and then centrifuged for ten minutes at 1500 rpm to collect particles on filters. After centrifugation, the supernatant solution was discarded and the pellet was inoculated onto 1.5% nonnutrient agar (NNA) plates. A dense suspension of heat inactivated *Escherichia coli*, prepared in Page Saline, was seeded onto NNA plates to grow FLA. After the inoculation of the samples, all plates were incubated at 30°C and examined daily for the presence of FLA for up to 10 days using a light microscope (100x). Once a growth was observed, a piece of NNA containing the amoebae was excised to inoculate a fresh NNA plate to subculture and incubated until trophozoites were grown. Then, the trophozoites were scraped to isolate genomic DNA (QIAmp DNA Mini Kit, QIAGEN).

**Figure 2:**
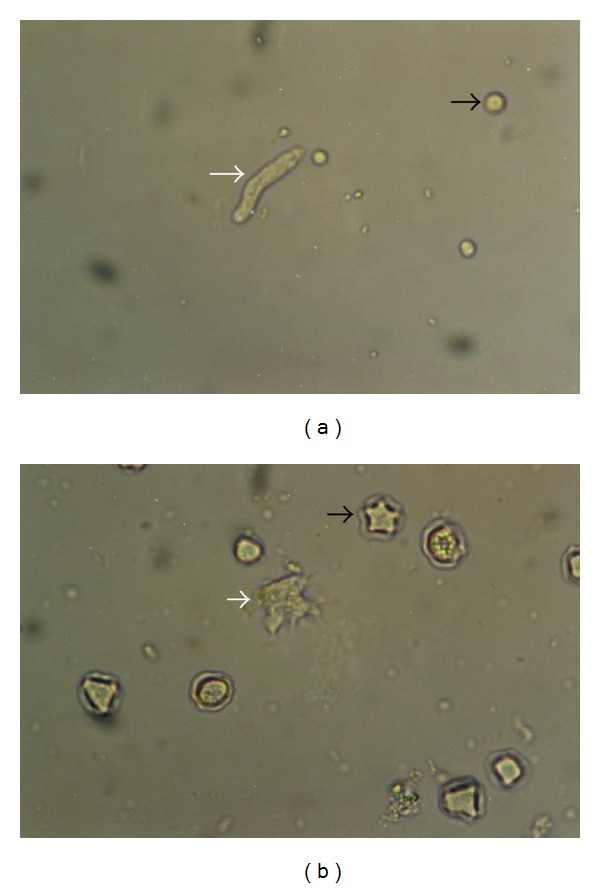
*Hartmannella *(a) and *Acanthamoeba *(b) trophozoites (white arrow), cyst forms (black arrow) (original magnification; 40x).

Amoebae were isolated and identified by morphologic features as well as PCR based sequence analysis. Smirnov and Goodkov's basic morphotyping list was used to identify amoebae [19]. *Acanthamoeba* was identified both by acanthopodia in the trophozoite form and by double-layered polygonal walls in the cysts form (Figure 2(a)). The *Hartmannella *was identified by smooth, spherical appearance (Figure 2(b)) [16, 20]. A temperature tolerance test was also performed for *Acanthamoeba*: three sets of subculture plates (NNA-*E. coli*) for each sample were incubated at 37, 42, and 52°C, respectively. All plates were examined daily for amoebal growth by phase contrast microscopy for seven days [21]. When an FLA strain was isolated, the flagellate transformation test was applied for the identification of *N. fowleri* [20]. All FLA strains were transferred to a fresh NNA-*E. coli* plate every month to check their viability and each of them were used in the experiments.

### 2.2. PCR Amplification, Sequencing, Blast Search of Sequenced Amplicons, and Cluster Analysis of Amoebae

*Acanthamoeba, N. fowleri, B. mandrillaris*,and* Sappinia* genus specific primer pairs along with common amoebae specific primers (Table 1) were employed in molecular detection of the amoebae species [7, 10, 22–24]. Fifty *µ*L of PCR mixture contained 1 ng DNA, 5 *µ*L 10x Taq buffer, 5 *µ*L 2 mM dNTP, 4 *µ*L 25 mM MgCl_2_, 0.5 *µ*L 100 mM primer, 0.5 *µ*L Taq DNA Polymerase. The thermal cycling conditions were an initial incubation of 94°C for 7 min and 45 cycles of 94°C for 60 s (95°C for *Acanthamoeba*), X°C for 60 s, and 72°C for 60 s with a terminal extension of 72°C for 10 min (X = 55°C for *Naegleria*, *Sappina*, and *Balamuthia*; 60°C for *Acanthamoeba*; and 65°C for common primers). PCR reactions were performed to amplify 18S rDNA sequences. These primers yielded 750–1000 bp fragments. The PCR amplicons were separated by 1% agarose gel (data not shown but available on request). Amplicon sizes were estimated using a DNA ladder. Amplicons were gel purified prior to sequencing. Sequencing was unidirectional (5-GTCAGAGGTGAAATTCTTGG-3′). Nucleotide similarity search was performed by blast search (basic local alignment search tool) of sequenced amplicons against amoeba species. *Acanthamoeba* has been classified into genotypes based on 18S ribosomal RNA nucleotide sequencing recently [25, 26]. GenBank accession numbers of these species are given in Table 2. Morphological observations and sequencing yielded three different species: *A. castellanii, Hartmannella vermiformis, *and *A. polyphaga*.

**Figure 3:**
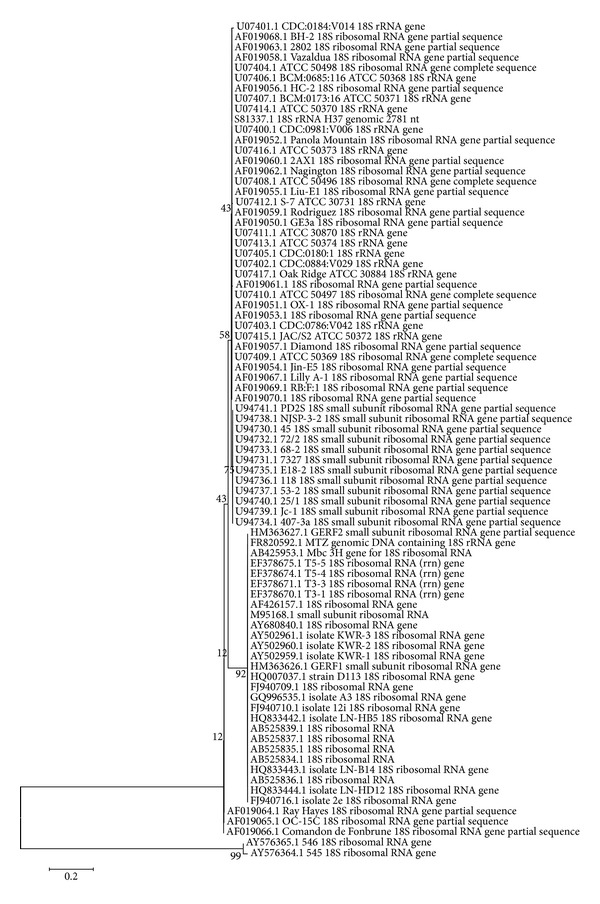
Phylogenetic analysis of FLA isolates. Neighbor-joining tree based on 18S rDNA sequences. The sequences from Sivas isolates were aligned by MEGA software using reference isolates from GenBank. Bar is index of evolutionary distances (0.2) among the different sequences. The numbers on the branch nodes of phylogenetic tree corresponded to bootstrap value.

Cluster analysis was performed for FLA by using the latest version of MEGA 5.05 software [27]. Phylogenetic construct was produced by employing 18S rRNA gene sequences and amoebae genus 18S rDNA gene sequences accession numbers listed in Table 2. These were aligned with the corresponding sequences from 33 reference *Acanthamoeba* isolates, and a neighbor-joining tree was obtained using MEGA 5.05 (Figure 3) [27].

## 3. Results

FLA were detected in 33 (22%) out of 150 water samples in six Sivas districts. All *Hartmannella *isolates (*n* = 24) were identified as *H. vermiformis* and except one isolate, which was identified as *A. polyphaga*, all *Acanthamoeba *isolates (*n* = 8) were identified as *A. castellanii*. No representative of any of the other three genera of FLA of clinical relevance, *Balamuthia, Naegleria*, and *Sappinia,* was present in any of the samples (Table 2). It was observed that all *Acanthamoeba* isolates were capable of growth at all incubation temperatures and they grew easily and fast at 37°C incubation but the amoeba trophozoites grew slower at either 42°C or 52°C of incubations. After two days of incubation, respective *Acanthamoeba* isolates stayed alive at 42°C (samples numbers, 2, 13, 19, 27, 30, 32, and 33) and at 52°C (samples numbers, 2, 13, 27, 30, 32, and 33). The flagellate transforming test was found to be negative for all the 33 isolates.

Morphological identification of amoebae revealed *Acanthamoeba *and *Hartmannella *trophozoites. *Acanthamoeba *strains belong to the T4 genotype as confirmed by genus specific primer pair (Table 1).

The main target of the current study was to apply and evaluate molecular methods to recognize FLA along with classical microscopic determination method. We used PCR primers to diagnose FLA species by employing a general primer set and four genus specific primer sets (Table 1). In this study FLA isolates were compared to GenBank and the reference strains (Table 2) to determine species. PCR amplicon lengths varied from 500 to 1000 base pairs. 

*Acanthamoeba* isolates were further examined by phylogenetic analyses by comparison of sequenced amplicons to *Acanthamoeba* strains. This included all representative sequences available from GenBank. All *Acanthamoeba* isolates were clustered in sequence type group T4. 

A homology search was performed with BLAST from NCBI. The deduced sequences were aligned by ClustalW with FLA sequences and the phylogenetic tree was then displayed by neighbor-joining analysis conducted with MEGA (Figure 3). All sequences gave 100% similarity except sample number 2 (99%) with accession numbers being given in Figure 3. In the phylogenetic tree *Acanthamoeba *and *Hartmannella* species clustered in different branches but sequences of the same genus clustered together in the same branch. This shows their genetic similarity to a certain degree and consistency with identification of isolated samples. 

Neighbor-joining gene tree analysis identifies individual strains of the isolates obtained in this study as revealed by reference numbers given at Table 2. The phylogenetic analysis illustrated that *Acanthamoeba *isolates were clustered to pathogenic genotype T4 and closest to U07413 reference number. However, two *Acanthamoeba* isolates belong to the U7413 and U07407 reference numbers. Similarly, *Hartmannella *isolates belong to the AF426157 reference number. 

The phylogenetic analysis confirms clinically relevant amphizoic amoebae in tap water which may present a risk to people's health. 

## 4. Discussion

FLA were distributed worldwide and the composition of these species at certain locations depends on the surroundings. Also, the spreading of FLA species depends on its tolerance to survive under adverse conditions. Therefore, the ecological importance of FLA must be adequately studied to prevent fatal human diseases. In this present comprehensive study, waterborne amoebae were isolated in various districts of Sivas province. We found FLA nearly in one out of five water sources in Sivas districts. Results revealed that *Acanthamoeba *and *Hartmannella* have a high distribution in the samples compared to other FLA. Although specific primers were designed, we did not isolate any of *N. fowleri, B. mandrillaris*,* and Sappinia *sp. during the study. This could be due to a lower prevalence of such FLA in the environment. Our findings are in agreement with a study from Bulgaria [7]. This Bulgarian study group determined *Acanthamoeba *and *Hartmannella *abundantly in water and soil samples compared to other species in Bulgaria. The high prevalence of the above FLA in human-related habitats was observed in environmental freshwaters of several countries as reported by the studies [28, 29]. 

*Acanthamoebae* have been isolated previously from bottled drinking water, tap water, soil, and dust in Burdur and İstanbul provinces in Turkey [15]. Furthermore both *Acanthamoebae* and *Naegleria* have also been isolated from soil and thermal water specimens in our region [16]. However, in that study, the amoebae have not been identified below the genus level. In our study except *Naegleria* we have also shown the presence of *Acanthamoeba* in the same region and identified those species. *Acanthamoeba* isolates belonging to T2, T3, T4, and T7 genotypes from Ankara [17] and T4 and T9 genotypes from Aydin province [13] have been found in environmental samples in Turkey.

To our knowledge the presence of FLA in the environment does not mean a risk factor for illness. However, many species of *Acanthamoeba *are potentially pathogenic for animals and for humans. *A. castellanii, A. polyphaga*, and* A. culbertsoni *are the most common species to infect humans [30, 31]. *Acanthamoeba *spp. are the main cause of AK associated with contact lenses [2, 3], although cases involving* Hartmannella *sp. have also been described [32].* H. vermiformis *has been suggested as a cause of AK but this is still under debate by other investigators [33]. In a study performed in Aydin province, in the western part of Turkey, a case of AK caused by a genotype T4* Acanthamoeba* strain related possibly with source of tap water was reported [13]. Another genotype, T4 *Acanthamoeba* strain, was also reported in an AK case in İzmir province, a neighbor province of Aydin. Tap water has been assumed to be the most important source of AK caused by genotype T4 *Acanthamoeba* [7] and worldwide the most important AK-causing strains are associated with genotype T4 [34, 35].

*Acanthamoeba *can tolerate extreme temperatures and thus become cold resistant, *A. polyphaga *may survive below 4°C. Some strains of *Hartmannella *can tolerate up to 48°C [11, 36]. Since Sivas province is a place of extreme hot and cold temperatures during the year, we conclude that determined FLA species are tolerant to ecological conditions. Air temperature ranges between winter and summer have been reported as −34.6 to 40°C during the years of 1972 to 2011 in Sivas (Turkish State Meteorological Service at http://www.dmi.gov.tr). In addition to environmental temperature conditions, several other factors such as cyst structure and surface availability of the organism, pH alterations, and osmolarity changes in water also determine the FLA life [37].

*Acanthamoeba *spp.* and Hartmannella *spp. can harbor pathogenic microorganism which indicates the importance of these amoebae to public health. *Acanthamoeba *T4 genotype and *H. vermiformis *may be infected naturally by pathogenic ARMs. *Acanthamoeba *T4 genotype may be infected by *Legionella *sp. and* Neochlamydia *sp., whereas *H. vermiformis* may be infected by *Neochlamydia *sp. and* Legionella donaldsonii* [37]. FLA can facilitate growth and transportation of waterborne pathogens. Therefore, they are used by pathogenic waterborne ARMs to proliferate in drinking water systems. We have limited data about host FLA in drinking water. It was reported previously that the infected FLA rate by ARM in drinking water system was 16% [5]. It is likely that several other infected FLA by unidentified pathogenic ARMs are yet to be determined.

Interestingly, FLA detection reported the presence of several genera of FLA, namely, *Acanthamoeba*, *Naegleria*, and *Hartmannella, *at different stages of the water treatment in drinking water treatment plants [38, 39]. Furthermore, *Hartmannella* spp. have been reported to resist disinfection in treatment plants [38, 39]. *Acanthamoeba *spp. can resist water treatment for sanitizing drinking water as well [28]. This data is consistent with our findings since 24 out of 33 detected FLA were *H. vermiformis *and 9 out of 33 detected FLA were *Acanthamoeba *spp. (Table 2). One other reason is that the high *H. vermiformis* prevalence in our region might be due to high levels of active biomass and natural organic matter [40] since agriculture and animal husbandry is common in Sivas province. 

## 5. Conclusions

The results indicated the presence of *A. castellanii, H. vermiformis, *and *A. polyphaga *in tap water in Sivas localities. Furthermore, presence of temperature tolerant *Acanthamoeba* T4 genotypes in the region must be taken into account for AK. 

FLA were underestimated by medical community until some species of amebae caused systemic infections in immunocompromised individuals. The amebic diseases are difficult to determine clinically and a patient may suffer from delay in treatment. Further, this delay may lead to deadly infections and cause fatal cases. Therefore, an investigation of the connection between environment and the patient's infections is essential. An individual history including interaction with amebic water and inhalation of cysts during dust storm may help a physician to diagnose the infection. Knowledge of prior prevalence of amebae in the region may help physicians to diagnose and treat either healthy or immunocompromised individuals. Clinicians may benefit from the reported data in the treatment of amebae related infections. Presence of FLA can lead to take precautions and reported FLA distribution can help understand the potential threat to the health of individuals.

The data obtained from the study may be beneficial for the clinicians and the environmental professionals in the region and regions around the world that have similar ecological conditions.

## Figures and Tables

**Table 1:** Primers used in this study (5′ → 3′).

Species	Forward primer	Reverse primer	Reference
Common for FLA	CGCGGTAATTCCAGCTCCAATAGC	CAGGTTAAGGTCTCGTTCGTTAAC	Tsvetkova et al., 2004 [7]
*Acanthamoeba *spp*. *	GGCCCAGATCGTTTACCGTG	TCTCACAAGCTGCTAGGGAGTCA	Schroeder et al., 2001 [22]
*N. fowleri *	CAAACACCGTTATGACAGGG	CTGGTTTCCCTCACCTTACG	Schild et al., 2007 [23]
*B. mandrillaris *	CGCATGTATGAAGAAGACCA	TTACCTATATAATTGTCGATACCA	Booton et al., 2003 [24]
*Sappinia *	TCT GGT CGC AAG GCT GAA AC	GCA CCA CCA CCC TTG AAA TC	Qvarnstrom et al., 2009 [10]

FLA: Free-living amoebae.

**Table 2:** Species, genotypes, GenBank accession numbers, and isolation sources of free living amoebae in districts of Sivas, Turkey.

No.	Species, identified	Genotype	Gene Bank ref. no.	Source of tap water
1	*Acanthamoeba castellanii *	T4	U07403	Divriği village
2	*Acanthamoeba castellanii *	T4	U07413	Kangal village
3	*Hartmannella vermiformis *		AF426157	Suşehri village
4	*Hartmannella vermiformis *		AF426157	Kangal village
5	*Hartmannella vermiformis *		AF426157	Kangal village
6	*Hartmannella vermiformis *		AF426157	Kangal village
7	*Hartmannella vermiformis *		AF426157	Kangal city center*
8	*Hartmannella vermiformis *		AF426157	Suşehri village
9	*Hartmannella vermiformis *		AF426157	Suşehri village
10	*Hartmannella vermiformis *		AF426157	Kangal village
11	*Hartmannella vermiformis *		AF426157	Suşehri village
12	*Hartmannella vermiformis *		AF426157	Divriği village
13	*Acanthamoeba castellanii *	T4	U07413	Kangal village
14	*Hartmannella vermiformis *		AF426157	Ulaş village
15	*Hartmannella vermiformis *		AF426157	Suşehri village
16	*Hartmannella vermiformis *		AF426157	Divriği village
17	*Hartmannella vermiformis *		AF426157	Gölova village
18	*Hartmannella vermiformis *		AF426157	Divriği village
19	*Acanthamoeba castellanii *	T4	U07413	Kangal village
20	*Hartmannella vermiformis *		AF426157	Kangal village**
21	*Acanthamoeba castellanii *	T4	U07413	Kangal village
22	*Acanthamoeba castellanii *	T4	U07413	Divriği hospital
23	*Hartmannella vermiformis *		AF426157	Suşehri village
24	*Hartmannella vermiformis *		AF426157	Ulaş healthcare center
25	*Hartmannella vermiformis *		AF426157	Gemerek village
26	*Hartmannella vermiformis *		AF426157	Kangal village
27	*Hartmannella vermiformis *		AF426157	Kangal village
28	*Hartmannella vermiformis *		AF426157	Suşehri village
29	*Hartmannella vermiformis *		AF426157	Ulaş village
30	*Acanthamoeba castellanii *	T4	U07413	Kangal village
31	*Hartmannella vermiformis *		AF426157	Suşehri village
32	*Acanthamoeba polyphaga *	T4	U07407	Ulaş village
33	*Acanthamoeba castellanii *	T4	U07413	Kangal village

*Spring water.

**Fountain water.
